# The association between rs16917496 T/C polymorphism of *SET8* gene and cancer risk in Asian populations: a meta-analysis

**DOI:** 10.1042/BSR20180702

**Published:** 2018-11-14

**Authors:** Hui-Xia Wei, Guo-Xiang Tian, Ju-Kun Song, Lian-Jie Yang, Yu-Pei Wang

**Affiliations:** 1 Department of Anesthesiology and Stomatology, Taihe Hospital, Hubei University of Medicine, Shiyan 442000, China; 2 Department of 4th Cadres Ward, PLA Army General Hospital, Beijing100700, China; 3 Department of Oral and Maxillary Surgery, Guizhou Provincial People’s Hospital, Guiyang 550002, China; 4 Trade union, Taihe Hospital, Hubei University of Medicine, Shiyan 442000, China

**Keywords:** cancer, miR-502, polymorphism, rs16917496, SET8

## Abstract

Epidemiological studies have demonstrated close associations between *SET8* rs16917496 T/C polymorphism and cancer risk, but the results of published studies were not consistent. We therefore performed this meta-analysis to explore the associations between rs16917496 T/C polymorphism and cancer risk. Five online databases were searched. Odds ratios (ORs) with a 95% confidence interval (CI) were calculated to assess the association between rs16917496 T/C polymorphism and cancer risk. In addition, heterogeneity, accumulative, sensitivity analysis, and publication bias were conducted to check the statistical power. Overall, 13 publications involving 5878 subjects were identified according to included criteria. No significant cancer risk was observed in genetic model of *SET8* rs16917496 T/C polymorphism in Asian populations (C vs. T: OR = 1.04, 95%CI = 0.88–1.23, *P* = 0.63%; TC vs. TT: OR = 1.17, 95%CI = 0.96–1.24, *P* = 0.11%; CC vs. TT: OR = 0.90, 95%CI = 0.60–1.37, *P* = 0.63; TC+CC vs. TT: OR = 1.11, 95%CI = 0.90–1.38, *P* = 0.33; CC vs. TT+TC: OR = 0.92, 95%CI = 0.65–1.30, *P* = 0.63). Furthermore, similar associations were found in the subgroup analysis of race diversity, control design, genotyping methods, and different cancer types. In summary, our meta-analysis indicated that the *SET8* rs16917496 T/C polymorphism may not play a critical role in cancer development in Asian populations.

## Introduction

Cancer is one of the most common causes of death, second only to cardiovascular disease [1]. In 2012, more than 14.1 million people suffered from cancer and 8.2 million people died from the disease worldwide. In China, approximately 4,292,000 new cancer cases and 2,814,000 cancer deaths were recorded in 2015 [2]. Surgery, radiotherapy, and chemotherapy are the most important cancer treatments [3–5]. However, these methods induce harmful side effects in patients, and the resulting damage to the treated tissues and organs causes considerable physical dysfunction as well as a reduction in quality of life. As a result, cancer and its complications are major mental and economic burdens for patients and their families. To date, the etiology and pathogenesis of cancer have been extensively explored, but the underlying mechanisms remain unclear [6,7]. Prior studies indicate that environmental pollution [8], unhealthy living habits [9], viral infection [10], and radioactive exposure [11] may contribute to tumorigenesis.

The diversity in cancer susceptibility within different populations has also not yet been explained. Molecular studies have proposed that genetic factors may play an important role in tumorigenesis [12,13]. One potential genetic factor, SET domain-containing protein 8 (*SET8*), encodes H4 lysine 20 monomethyltransferase, which is present in mitotic cell chromosomes and belongs to the family of methyltransferases contained in the SET domain. *SET8* could be involved in multiple biological processes, such as gene transcriptional regulation, genome replication genomic stability, and cell cycle management [14,15]. Many studies have indicated that increased expression of *SET8* can suppress the transcriptional activation of the p53 protein and inhibit cell apoptosis. It also promotes tumor invasion and migration by enhancing the epithelial–mesenchymal transition (EMT) [16,17]. The aberrant expression or dysfunction of *SET8* may affect cell cycle progression and alter an individual’s cancer risk, resulting in a close association between *SET8* and the risks of various cancers [18,19]. In addition, some studies have reported that *SET8* is a target gene of many miRNAs during the development of cancer, including miR-502, miR-382, miR-335, miR-127-3p, and miR-7. Ma found that *SET8* expression was upregulated in glioma cells and that there is a significant inverse correlation between SET8 expression and miR-382 [20]. Zhang et al. reported that *SET8* was directly regulated by miR-127-3p, with miR-127-3p overexpression resulting in decreased expression of *SET8* and reduced migration and invasion of osteosarcoma cells [21]. Yu et al. also observed that miRNA-7 is a negative regulator of *SET8* and further inhibits H4K20 monomethylation to suppress EMT and the invasive potential of breast cancer cells [22]. This evidence suggests that *SET8* may be a potential molecular target for the prevention and treatment of cancers.

Single-nucleotide polymorphisms (SNPs) are the most common genetic variations in the human genome. The rs16917496 T/C polymorphism is the most important variant locus located in sequences of the 3’-untranslated region (UTR) of the *SET8* mRNA, which influences reorganization and binding to miR-502. In 2009, Song et al. reported the first case–control study in a Chinese population with breast cancer; results suggest that the miR-502 binding site (rs16917496 T/C polymorphism) of *SET8* contributes to an increased breast cancer risk at an early age [23]. Since then, a series of epidemiological studies have been conducted to investigate the association between the rs16917496 T/C polymorphism and cancer risk; however, the results are inconsistent. Therefore, we conducted this meta-analysis to more precisely and comprehensively assess the association between the *SET8* rs16917496 T/C polymorphism and cancer risk.

## Materials and methods

This meta-analysis was designed and performed according to the guidelines of the preferred reporting items for systematic reviews and meta-analyses compliant statement [24]. All included data were collected from published studies and no ethical issues were involved.

### Search strategy

We searched three English databases (PubMed, Embase, and Science Citation Index) and two Chinese databases (CNKI and Wanfang) for relevant studies that were conducted prior to December 1, 2017 and investigated the association between *SET8* rs16917496 T/C polymorphism and cancer risk. The bibliographies of the included studies were reviewed to identify additional studies. The following search terms and strategy were used (e.g. in PubMed):

#1 SET8

#2 SETD8

#3 microRNA-502

#4 miR-502

#5 rs16917496

#6 #1 OR #2 OR #3 OR #4 OR #5

#7 polymorphism

#8 variant

#9 mutation

#10 #7 OR #8 OR #9

#11 cancer

#12 tumor

#13 neoplasm

#14 #11 OR #12 OR #13

#15 #6 AND #10 AND #14

### Eligibility criteria

Studies were selected according to the following criteria: (1) they should be case–control studies that investigated the association between *SET8* rs16917496 T/C polymorphism and cancer risk; (2) studies should provide sufficient data on genotype distribution which could be used to evaluate odds ratios (ORs) and 95% confidence intervals (CIs); (3) studies were published either in English or Chinese; and (4) publications included the largest or most recent sample data available when overlapping or duplicate data were published on the same item.

### Data extraction

Two authors (Wei and Wang) reviewed the qualified studies and extracted the following information: the name of the first author, publishing date, country or region of the subjects, race of the subjects, control design, sample sizes, genotype distribution of cases and controls, genotyping method, and cancer type. Modified Newcastle–Ottawa scale (NOS) was employed by the first two authors in order to evaluate the quality of the included studies according to the publication of Niu et al. [25]. The scores ranged from 0 (worst) to 10 points (best). Studies with a score of 7 or higher were classified as high quality (Table 3).

### Statistical analysis

Crude ORs and 95% CIs were calculated to evaluate the association between *SET8* rs16917496 T/C polymorphism and cancer risk. Hardy–Weinberg equilibrium (HWE) was assessed in the controls by using genotype frequencies of the *SET8* rs16917496 T/C polymorphism with the χ^2^ test. Five genetic models, namely allele contrast (C vs. T), co-dominant (TC vs. TT and CC vs. TT), dominant (TC+CC vs. TT), and recessive models (CC vs. TT+TC) were used. Heterogeneity was assessed among the included studies by using Cochran’s Q test and *I*^2^ statistical method [26]. The random-effect model (DerSimonian and Laird method) was adopted when the *I*^2^ value was greater than 40%; otherwise, the fixed-effect model (Mantel–Haenszel method) was applied [27,28]. Subgroup analysis was conducted on HWE status, race, control design, sex, genotyping method, and cancer type. Meta-regression analysis was performed using the genetic modes to determine between-group heterogeneity. Cumulative and sensitivity analyses were conducted to examine possible changes when single studies were omitted. Potential publication bias was assessed with Egger’s linear regression and Begg’s funnel plots [29,30]. All statistical analyses were conducted with STATA version 14.0 (Stata Corporation, College Station, TX, U.S.A.). A two-sided *P*<0.05 was considered statistically significant.

## Results

### Study characteristics

**Figure 1:**
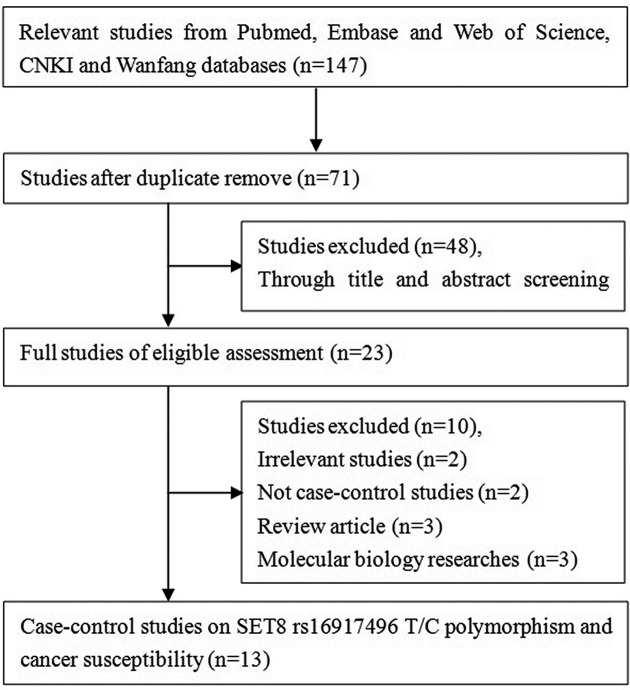
Flow diagram of the study selection process

Figure 1 provides a schematic representation of the selection process. A total of 147 articles were identified through online search; of these, 76 studies were excluded due to article duplication, and 48 studies were excluded after the abstracts and full texts were reviewed. Finally, 13 studies involving 2866 patients and 3012 controls were included in our meta-analysis [23,31–42]. All of the subjects in these thirteen studies were from Asian countries, including nine case–control studies involving 2212 cases and 2314 controls conducted in Chinese populations [23,31–35,37,38,41], and four case–control studies involving 654 cases and 698 controls conducted in Iranian populations [36,39,40,42]. Population-based controls were used in seven studies [23,31–33,35–37] and hospital-based controls were used in six studies [34,38–42]. Seven studies used the polymerase chain reaction-restriction fragment length polymorphism method [23,35–37,39,40,42], five studies used the polymerase chain reaction-restriction sequencing method [31,32,34,38,41], and one study used the polymerase chain reaction-ligase detection method [33]. Furthermore, three studies focused on digestive system cancer [31,34,40], two studies on lung cancer [33,35], and two studies on ovarian cancer [32,41]. Only seven studies satisfied the HWE of the genotype distributions in the control groups. All characteristics of the included studies are presented in Table 1.

**Table 1:** Characteristics of included studies on *SET8* rs16917496 T/C polymorphism and cancer risk

First author	Year	Country /Region	Race	Source of controls	Case	Control	Genotype distribution						Genotyping methods	*P* for HWE^*^	MAF in control	Type	NOS
							Case			Control							
							TT	TC	CC	TT	TC	CC					
Song [23]	2009	China	East Asia	PB	1100	1079	504	491	115	518	475	104	PCR-RFLP	0.74	0.31	BC	9
Guo [31]	2012	China	East Asia	PB	133	142	72	50	11	73	55	14	PCR-sequencing	0.45	0.29	HC	8
Wang [32]	2012	China	East Asia	PB	342	344	160	155	27	167	132	45	PCR-sequencing	0.02	0.32	OC	6
Ding [33]	2012	China	East Asia	PB	44	42	22	14	8	24	12	6	PCR-LDR	0.05	0.29	LC	6
Zhao [34]	2013	China	East Asia	HB	65	60	32	25	8	30	26	4	PCR-sequencing	0.60	0.28	ESC	7
Yang [35]	2014	China	East Asia	PB	164	199	95	57	12	102	69	28	PCR-RFLP	0.01	0.31	LC	5
Hashemi [36]	2014	Iran	Western Asia	PB	75	115	3	59	13	0	108	7	PCR-RFLP	<0.01	0.53	ALL	7
Yang [37]	2014	China	East Asia	PB	114	200	44	42	28	111	63	26	PCR-RFLP	<0.01	0.29	CC	6
Zhang [38]	2017	China	East Asia	HB	140	130	79	47	14	68	32	30	PCR-sequencing	<0.01	0.35	RCC	6
Narouie [39]	2017	Iran	Western Asia	HB	169	182	29	94	46	65	83	34	PCR-RFLP	0.41	0.41	PC	8
Mosallayi [40]	2017	Iran	Western Asia	HB	170	170	58	80	32	69	71	30	PCR-RFLP	0.12	0.39	CRC	8
Li [41]	2017	China	East Asia	HB	100	100	43	52	5	49	38	13	PCR-sequencing	0.20	0.32	OC	7
Parchami Barjui [42]	2017	Iran	Western Asia	HB	240	231	38	183	19	21	172	38	PCR-RFLP	<0.01	0.54	BC	5

* HWE in control

Abbreviations: ALL, acute lymphoblastic leukemia; BC, breast cancer; CC, cervical cancer; CRC, colorectal cancer; ESC, esophageal cancer; HB, hospital-based; HC, hepatocellular cancer; LC, lung cancer; MAF, minor allele frequency in control group; OC, ovarian cancer; PB, population-based; PC, prostate cancer; PCR-LDR, polymerase chain reaction-ligase detection reaction; PCR-RFLP, created restriction site-restriction fragment length polymorphism; PCR-sequencing, polymerase chain reaction-sequencing; RCC, renal cell carcinoma.

### Quantitative and subgroup analyses

**Figure 2:**
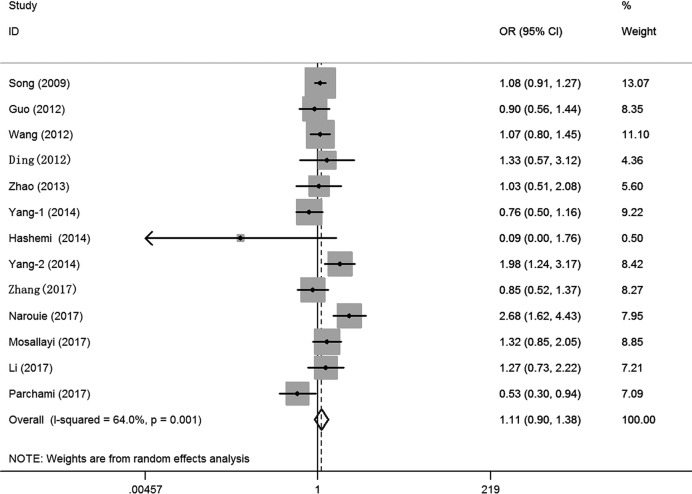
OR and 95% CIs of the associations between *SET8* rs16917496 T/C polymorphism and cancer risk in TC+CC vs. TT model

Overall, no significant association was observed between the *SET8* rs16917496 T/C polymorphism and cancer risk in all five genetic models in Asian populations (C vs. T: OR = 1.04, 95%CI = 0.88–1.23, *P* = 0.63, *I*^2^ = 72.3%; TC vs. TT: OR = 1.17, 95%CI = 0.96–1.24, *P* = 0.11, *I*^2^ = 50.0%; CC vs. TT: OR = 0.90, 95%CI = 0.60–1.37, *P* = 0.63, *I*^2^ = 76.9%; TC+CC vs. TT: OR = 1.11, 95%CI = 0.90–1.38, *P* = 0.33, I^2^ = 64.0%, (Figure 2); CC vs. TT+TC: OR = 0.92, 95%CI = 0.65–1.30, *P* = 0.63, *I*^2^ = 73.9%) (Table 2 and Supplementary Figure S1). Furthermore, no significant association was found between the *SET8* rs16917496 T/C polymorphism and cancer risk in the subgroup analysis of HWE status, race diversity, control design, and sex. Few protective effects of the *SET8* rs16917496 T/C polymorphism against cancer development were found in the PCR-sequencing subgroup (CC vs. TT: OR = 0.61, 95%CI = 0.44–0.86, *P* = 0.01, *I*^2^ = 18.6%; CC vs. TT+TC: OR = 0.59, 95%CI = 0.37–0.94, *P* = 0.03, *I*^2^ = 41.0%) and ovarian cancer subgroup (CC vs. TT: OR = 0.59, 95%CI = 0.37–0.94, *P* = 0.03, *I*^2^ = 0%; CC vs. TT+TC: OR = 0.52, 95%CI = 0.33–0.82, *P* = 0.01, *I*^2^ = 0%). Heterogeneity was observed in all five genotype models. Meta-regression analysis was conducted with all stratified factors, but no critical factors were found to contribute to heterogeneity (e.g. TC+CC vs. TT model: *P* = 0.23 for HWE status, *P* = 0.78 for race diversity, *P* = 0.82 for control design, *P* = 0.60 for sex, *P* = 0.81 for genotyping methods and *P* = 0.98 for cancer type).

**Table 2:** Summary ORs and 95% CI of *SET8* rs16917496 T/C polymorphism and cancer risk

	C vs. T					TC vs. TT				CC vs. TT				TC+CC vs. TT				CC vs. TT+TC			
	N*	OR	95% CI	*P*	*I*^2^ (%)	OR	95% CI	*P*	*I*^2^ (%)	OR	95% CI	*P*	*I*^2^ (%)	OR	95% CI	*P*	*I*^2^ (%)	OR	95% CI	*P*	*I*^2^ (%)
**Total**	13	1.04	0.88–1.23	0.63	72.3	1.17	0.96–1.24	0.11	50.0	0.90	0.60–1.37	0.63	76.9	1.11	0.90–1.38	0.33	64.0	0.92	0.65–1.30	0.63	73.9
**HWE status**																					
Yes	7	1.15	0.98–1.36	0.10	44.7	1.27	0.98–1.66	0.07	50.1	1.28	0.85–1.92	0.24	54.2	1.27	0.86–1.65	0.07	54.8	1.12	0.92–1.38	0.26	25.0
No	6	0.94	0.70–1.25	0.66	80.7	1.04	0.74–1.47	0.82	57.4	0.60	0.29–1.25	0.17	81.5	0.92	0.62–1.36	0.67	71.8	0.79	0.41–1.54	0.49	83.9
**Race**																					
China	9	1.00	0.83–1.20	0.98	66.9	1.12	0.99–1.27	0.08	0	0.87	0.57–1.34	0.53	70.6	1.07	0.95–1.20	0.25	29.1	0.82	0.54–1.24	0.34	71.5
Iran	4	1.14	0.77–1.68	0.51	83.7	1.07	0.47–2.42	0.88	82.4	0.90	0.27–2.99	0.86	87.3	1.04	0.44–2.48	0.93	85.5	1.19	0.57–2.47	0.64	82.0
**Design**																					
PB	7	1.06	0.87–1.30	0.56	68.1	1.09	0.95–1.24	0.22	20.8	0.97	0.60–1.55	0.89	68.2	1.08	0.85–1.36	0.52	52.5	1.08	0.68–1.69	0.75	72.6
HB	6	1.01	0.74–1.39	0.93	79.4	1.26	0.84–1.87	0.26	66.4	0.84	0.37–1.92	0.68	84.7	1.14	0.44–1.77	0.55	75.2	0.76	0.42–1.37	0.36	77.3
**Sex**																					
Female	5	1.04	0.81–1.33	0.70	79.6	1.15	0.88–1.49	0.31	55.9	0.79	0.41–1.55	0.50	84.7	1.11	0.83–1.48	0.49	68.6	0.78	0.44–1.37	0.39	82.3
**Genotyping**																					
PCR-RFLP	7	1.13	0.88–1.44	0.33	82.6	1.16	0.83–1.64	0.39	72.0	1.05	0.57–1.93	0.88	83.6	1.16	0.79–1.70	0.45	80.5	1.13	0.73–1.74	0.59	77.8
PCR sequencing	5	0.89	0.76–1.04	0.12	0	1.18	0.95–1.46	0.14	0	0.61	0.44–0.86	0.01	18.6	1.02	0.83–1.24	0.87	0	0.59	0.37–0.94	0.03	41.0
**Type**																					
DSC	3	1.07	0.86–1.33	0.54	0	1.18	0.79–1.48	0.63	0	1.16	0.73–1.85	0.53	0.0	1.09	0.81–1.46	0.57	0	1.08	0.70–1.66	0.73	0
BC	2	0.90	0.63–1.29	0.57	84.3	0.84	0.48–1.49	0.56	73.4	0.59	0.15–2.34	0.45	91.2	0.80	0.40–1.58	0.52	81.7	0.72	0.29–1.78	0.48	87.3
LC	2	0.90	0.51–1.58	0.71	60.8	0.95	0.63–1.42	0.79	0	0.74	0.24–2.24	0.59	60.9	0.89	0.55–1.42	0.63	24.9	0.72	0.27–1.91	0.51	53.8
OC	2	0.93	0.76–1.14	0.48	0.0	1.29	0.98–1.71	0.07	0	0.59	0.37–0.94	0.03	0.0	1.12	0.86–1.45	0.42	0	0.52	0.33–0.82	0.01	0

Abbreviations: DSC, digestive system cancer; HB, hospital-based; LC, lung cancer; OC, ovarian cancer; PB, population-based; PCR-RFLP, created restriction site-restriction fragment length polymorphism.

* Numbers of comparisons

**Table 3:** Scale for quality evaluation

Criteria	Score
**Representativeness of cases**	
Consecutive/randomly selected cases with clearly defined sampling frame	2
Not consecutive/randomly selected case or without clearly defined sampling frame	1
Not described	0
**Source of controls**	
Population- or Healthy-based	2
Hospital-based	1
Not described	0
**HWE in controls**	
HWE	2
Hardy–Weinberg disequilibrium	1
Not available	0
**Genotyping examination**	
Genotyping done under ‘blinded’ condition and repeated again	2
Genotyping done under ‘blinded’ condition or repeated again	1
Unblinded done or not mentioned and unrepeated	0
**Association assessment**	
Assess association between genotypes and cancer with appropriate statistics and adjustment for confounders	2
Assess association between genotypes and cancer with appropriate statistics and without adjustment for confounders	1
Inappropriate statistics used	0

### Cumulative and sensitivity analyses

**Figure 3:**
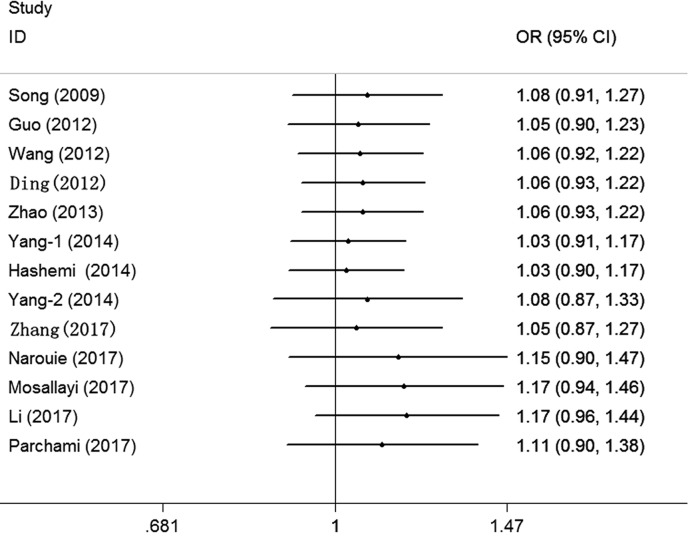
Cumulative meta-analyses according to publication year in TC+CC vs. TT model of *SET8* rs16917496 T/C polymorphism

**Figure 4:**
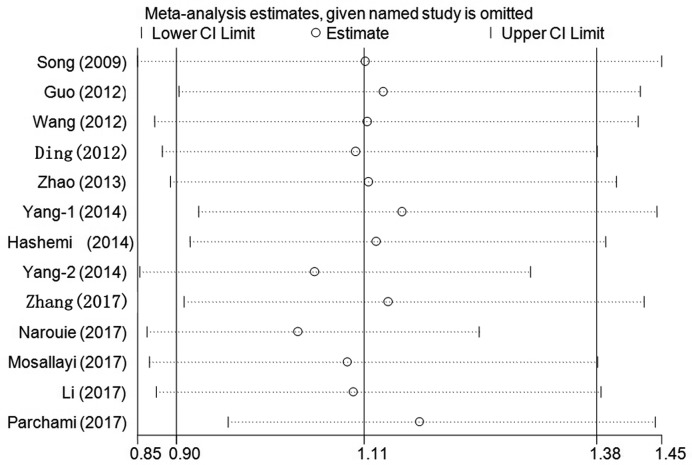
Sensitivity analysis through deleting each study to reflect the influence of the individual dataset to the pooled ORs in TC+CC vs. TT model of *SET8* rs16917496 T/C polymorphism

Cumulative analysis indicated a consistent and negative tendency accompanied by the continuously published studies (Figure 3 for TC+CC vs. TT model) (Supplementary Figure S2). Sensitivity analysis was performed by omitting each study one at a time, and the results did not indicate significant changes in the statistical analysis results (Figure 4 for TC+CC vs. TT model) (Supplementary Figure S3).

### Publication bias

**Figure 5:**
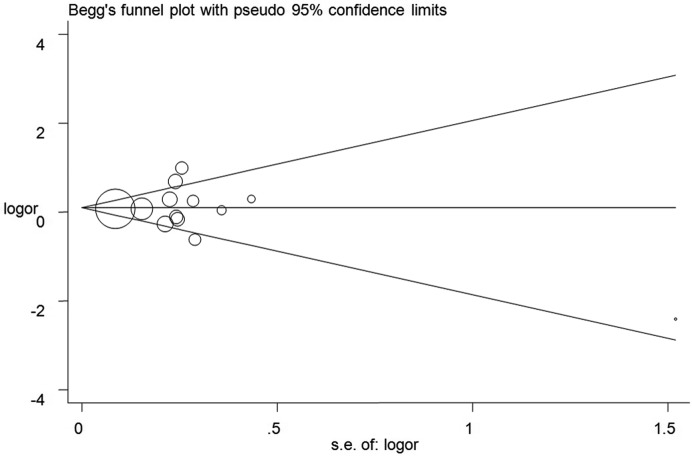
Funnel plot analysis to detect publication bias for TC+CC vs. TT model of *SET8* rs16917496 T/C polymorphism Circles represent the weight of the studies.

Publication bias was examined using the Begg’s test and no apparent asymmetry of the funnel plot was found (Figure 5 for TC+CC vs. TT model) (Supplementary Figure S4). These results were confirmed using Egger’s test (C vs. T: *P* = 0.90; TC vs. TT: *P* = 0.95; CC vs. TT: *P* = 0.48; TC+CC vs. TT: *P* = 0.83; CC vs. TT+TC: *P* = 0.77).

## Discussion

To date, cancer is still one of the most dangerous diseases worldwide. The improvement in cancer detection and treatments has resulted in decreased incidence and mortality rates of several cancer types. However, the etiology of cancer development, including both the molecular mechanisms and individual susceptibility, remains unclear.

MicroRNA (miRNA) is a highly conservative small non-coding RNA. It facilitates post-transcriptional mRNA expression and results in targeted mRNA degradation by base-paired binding to complementary sequences of the 3’-UTR of the target mRNA. SNPs, important variations in single-nucleotide sequences in a genome, contribute to genetic variations and influence the cancer susceptibility of individuals. SNPs in miRNA binding sites have been associated with cancer risk and outcomes. Alterations in these sites could modify the binding sites or change the binding affinity, leading to escape from miRNA regulation [43,44].

*SET8* (also known as *SETD8* or *PR-SET7*), located in chromosome 12q24.31, has various functions in multiple biological processes [45–47]. Rs16917496 T/C is the most common SNP locus located in the 3′-UTR of the *SET8* gene. Since 2009, several studies on SNPs and cancer risk have been published. Song et al. reported an increased risk in CC genotype that contributed to the early development of breast cancer (OR = 1.66, 95%CI = 106–2.61) [23]. Yang et al. [37] and Narouie et al. [39] reported that the C allele may increase the risk of cervical and prostate cancers in Chinese and Indian populations. In contrast, Yang et al. indicated that the TT genotype presents an increased lung cancer risk compared with the CC genotype in a Chinese population (OR = 2.17, 95%CI = 1.05–4.52) [35]. Moreover, Mosallayi et al. demonstrated the apparent association of increased colorectal cancer risk with the TT+TC genotypes based on smoking status among Turkish subjects (OR = 5.8, 95%CI = 1.37–24.34) [40]. However, several studies also reported a negative association between the rs16917496 T/C polymorphism and cancer risk [31,33,41]. All these results suggest that rs16917496 T/C may have an apparent linkage with carcinogenesis.

The contradictory results in the association of rs16917496 T/C polymorphism and cancer risk could be due to the following: (1) studied populations were from different countries and of different ethnicities; (2) different genotyping methods were used; (3) the genotype distributions in the controls in some studies deviate from HWE; and (4) control groups were not consistent. Therefore, we conducted this meta-analysis using the current 13 case–control studies involving 5878 subjects to assess the association between *SET8* rs16917496 T/C polymorphism and cancer susceptibility. To the best of our knowledge, the present study is the first meta-analysis that focused on *SET8* rs16917496 T/C polymorphism and cancer risk. The results showed no significant association between *SET8* rs16917496 T/C polymorphism and cancer susceptibility in Asian populations. A negative association was also found through analysis in the stratified groups. This meta-analysis utilized scientific retrieval strategy and rigorous statistic methodologies, such as cumulative, sensitivity, and meta-regression analyses.

The present study presents the following advantages: (1) it was the first and latest analysis that focused on the relationship between *SET8* rs16917496 T/C polymorphism and cancer risk and included comprehensive stratified analysis; (2) systematic and comprehensive retrieval strategies were conducted to collect all published studies carefully and avoid the omission of related research; (3) sensitivity, accumulation, and meta-regression analyses were conducted to identify factors affecting inconsistencies in the results; (4) Egger’s test and Begg’s funnel plots were conducted and did not detect any publication bias. However, the present work also presents several limitations that should be addressed in future studies: (1) only one SNP locus of rs16917496 T/C was investigated in this meta-analysis, and the interactions between relative polymorphism loci and gene-environment were not conducted with the current data; (2) the included sample size was small and widely distributed, which could influence the results and lead to incorrect conclusions; (3) subjects in the included studies were all Asian (from East Asia and West Asia), so ethnicity bias was inevitable and affected the results; (4) heterogeneity existed in some genetic models, and the subsequent meta-regression analysis could not identify interfering factors.

In summary, our analyses suggest that *SET8* rs16917496 T/C polymorphism is not a candidate genetic factor for cancer susceptibility. Considering the current limitations of this meta-analysis, further studies with larger sample sizes and participants of multiple ethnicities should be conducted to clarify the association between *SET8* rs16917496 T/C polymorphism and cancer risk.

## Supporting information

**Supplementary Figure S1:** OR and 95% CIs of the associations between *SET8* rs16917496 T/C polymorphism and cancer risk (A for C vs. T model; B for TC vs. TT model; C for CC vs. TT model; D for CC vs. TT+TC model).

**Supplementary Figure S2:** Cumulative meta-analyses according to publication year in *SET8* rs16917496 T/C polymorphism and cancer risk (A for A vs. G model; B for GA vs. GG model; C for AA vs. GG model; D for AA vs. GG+GA model).

**Supplementary Figure S3:** Sensitivity analysis through deleting each study to reflect the influence of the individual dataset to the pooled ORs in *SET8* rs16917496 T/C polymorphism and cancer risk (A for C vs. T model; B for TC vs. TT model; C for CC vs. TT model; D for CC vs. TT+TC model).

**Supplementary Figure S4:** Funnel plot analysis to detect publication bias in *SET8* rs16917496 T/C polymorphism and cancer risk (A for C vs. T model; B for TC vs. TT model; C for CC vs. TT model; D for CC vs. TT+TC model).

## Abbreviations

CI: confidence interval
EMT: epithelial–mesenchymal transition
HWE: Hardy–Weinberg equilibrium
NOS: Newcastle–Ottawa scale
OR: odds ratio
SNP: single-nucleotide polymorphism
UTR: untranslated region
