# Supplementary material for: The association between rs16917496 T/C polymorphism of SET8 gene and cancer risk in Asian populations: a meta-analysis
Source: Biosci Rep. 2018 Nov 14;38(6):BSR20180702. doi: 10.1042/BSR20180702 (PMC6239252; doi:10.1042/BSR20180702)

Supplementary Figure Legends

Supplementary Figure S1. OR and 95% CIs of the associations between *SET8* rs16917496 T/C polymorphism and cancer risk (A for C vs. T model; B for TC vs. TT model; C for CC vs. TT model; D for CC vs. TT+TC model).

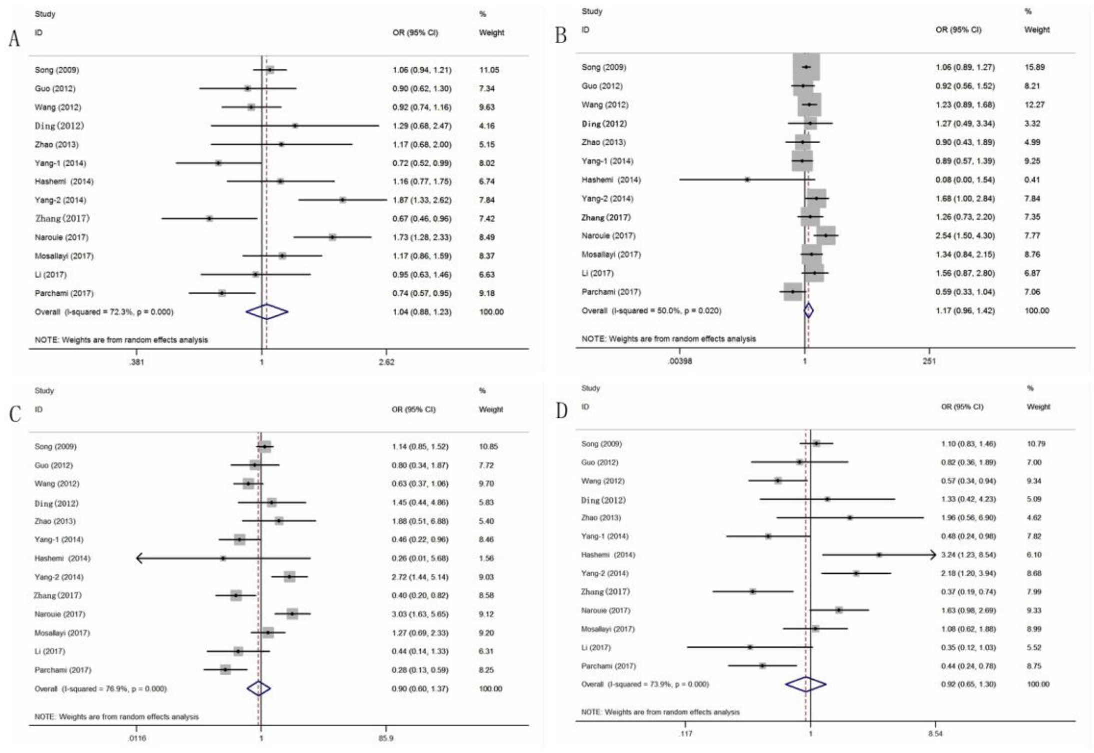

Supplementary Figure S2 Cumulative meta-analyses according to publication year in *SET8* rs16917496 T/C polymorphism and cancer risk (A for A vs. G model; B for GA vs. GG model; C for AA vs. GG model; D for AA vs. GG+GA model).

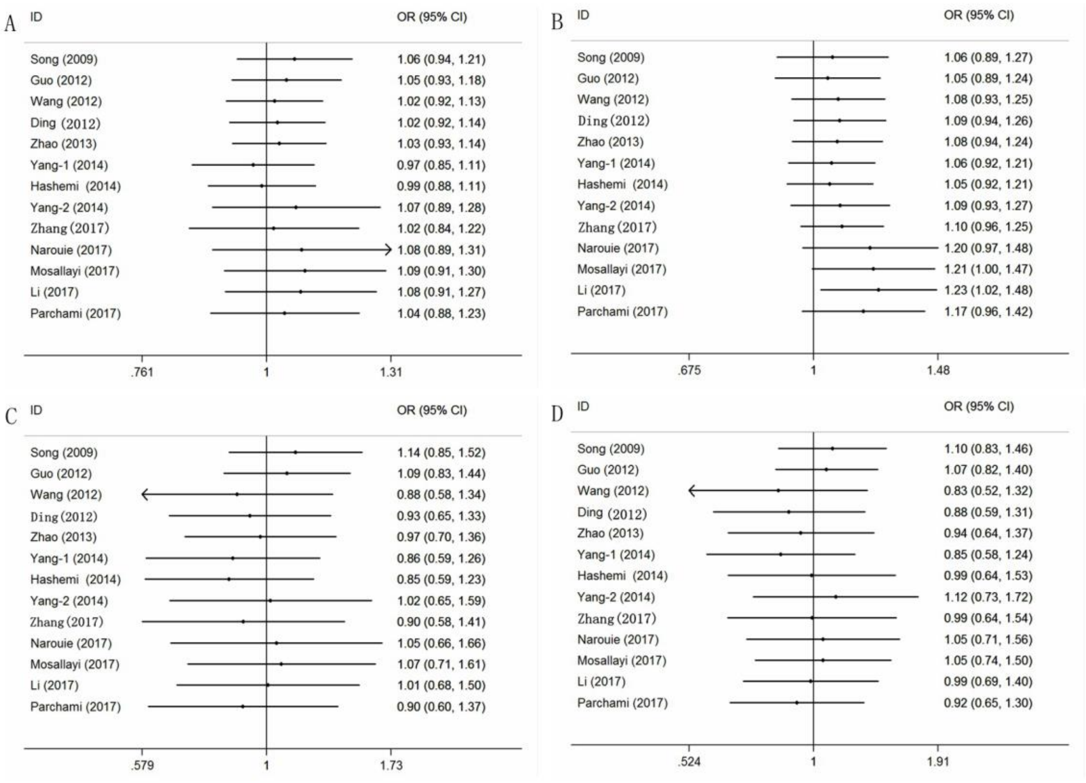

Supplementary Figure S3. Sensitivity analysis through deleting each study to reflect the influence of the individual dataset to the pooled ORs in *SET8* rs16917496 T/C polymorphism and cancer risk (A for C vs. T model; B for TC vs. TT model; C for CC vs. TT model; D for CC vs. TT+TC model).

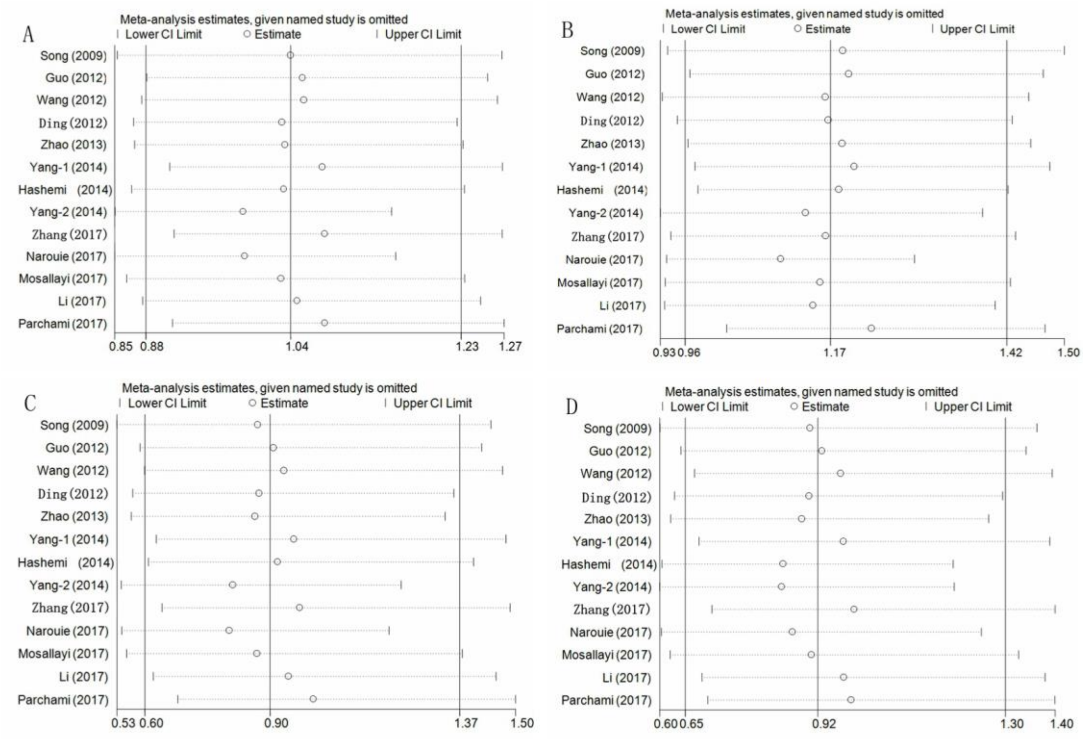

Supplementary Figure S4. Funnel plot analysis to detect publication bias in *SET8* rs16917496 T/C polymorphism and cancer risk (A for C vs. T model; B for TC vs. TT model; C for CC vs. TT model; D for CC vs. TT+TC model).

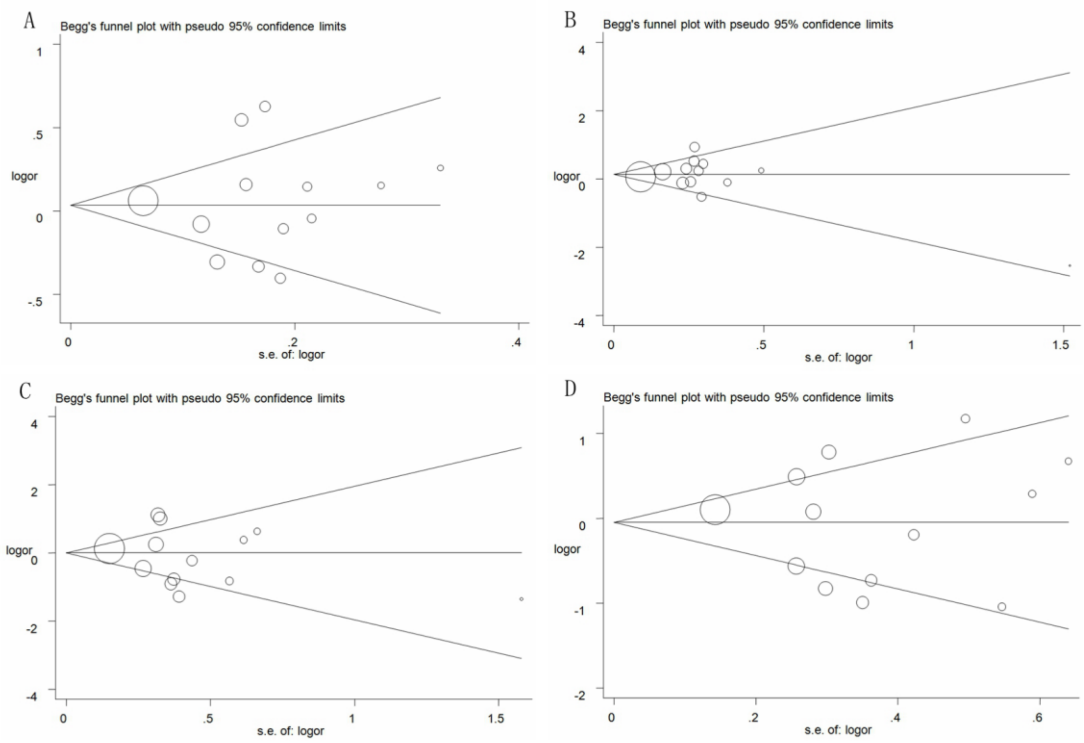

Supplement: Supplementary file 1 [file bsr20180702_Supp1.pdf]
